# DiscoVR: results of a multicenter RCT on a social cognitive virtual reality training to enhance social cognition in psychosis

**DOI:** 10.1192/j.eurpsy.2022.330

**Published:** 2022-09-01

**Authors:** G. Pijnenborg, S. Nijman, W. Veling

**Affiliations:** 1 GGZ Drenthe, Department Of Psychotic Disorders, Assen, Netherlands; 2 GGZ Drenthe, Dept Of Psychotic Disoders, Assen, Netherlands; 3 Uniersity Medical Center Groningen, Universitair Centrum Psychiatrie, Groningen, Netherlands

**Keywords:** virtual reality, social cognition, Psychosis, Treatment

## Abstract

**Introduction:**

Functional deficits, that is, problems in fulfilling appropriate social roles in daily life, are very common in people with a psychotic disorder. In recent years, Virtual Reality (VR) has emerged as a potential tool to improve SCT. Our research group has developed an immersive VR-SCT (‘Dynamic Interactive Social Cognition Training in Virtual Reality’: ‘DiSCoVR’).

**Objectives:**

To evaluate to effects of a VR-based social cognition training (SCT) for people with a psychotic disorder.

**Methods:**

This intervention was compared the an active VR-control condition in a multicenter RCT. Both interventions contained sixteen individual 45-60-minute on-site sessions, administered twice a week. Main study outcomes are social cognition and social functioning in daily life assessed with experience sampling.

**Results:**

From baseline to post-treatment (n=72), none of the time*group interactions were significant, indicating an absence of treatment effects. A significant effect of time was observed for the SERS total score (b=9.84, 95% CI=3.81-15.87, p=.002), indicating overall improvement in self-esteem.

**Conclusions:**

We did not find any significant treatment effects. An effect of time on self-esteem was found at post-treatment, but not follow-up, suggesting a temporary improvement in self-esteem in both groups. One way to interpret these results is that, contrary to other SCT interventions, DiSCoVR does not improve social cognition or social functioning. This could be due to characteristics of the treatment protocol. Another possibility is that, contrary to the premise of VR-SCT, our VR environments inadequately simulated reality. Adapting an established protocol to VR, could further elucidate the merit of VR as a training method.

**Disclosure:**

No significant relationships.

